# An intercalation-locked parallel-stranded DNA tetraplex

**DOI:** 10.1093/nar/gkv033

**Published:** 2015-01-27

**Authors:** Shailesh Tripathi, Daoning Zhang, Paul J. Paukstelis

**Affiliations:** Department of Chemistry & Biochemistry, Center for Biomolecular Structure & Organization, Maryland Nanocenter, University of Maryland, College Park, MD 20742, USA

## Abstract

DNA has proved to be an excellent material for nanoscale construction because complementary DNA duplexes are programmable and structurally predictable. However, in the absence of Watson–Crick pairings, DNA can be structurally more diverse. Here, we describe the crystal structures of d(ACTCGGATGAT) and the brominated derivative, d(AC^Br^UCGGA^Br^UGAT). These oligonucleotides form parallel-stranded duplexes with a crystallographically equivalent strand, resulting in the first examples of DNA crystal structures that contains four different symmetric homo base pairs. Two of the parallel-stranded duplexes are coaxially stacked in opposite directions and locked together to form a tetraplex through intercalation of the 5′-most A–A base pairs between adjacent G–G pairs in the partner duplex. The intercalation region is a new type of DNA tertiary structural motif with similarities to the i-motif. ^1^H–^1^H nuclear magnetic resonance and native gel electrophoresis confirmed the formation of a parallel-stranded duplex in solution. Finally, we modified specific nucleotide positions and added d(GAY) motifs to oligonucleotides and were readily able to obtain similar crystals. This suggests that this parallel-stranded DNA structure may be useful in the rational design of DNA crystals and nanostructures.

## INTRODUCTION

The ability for DNA oligonucleotides with complementary sequences to recognize each other in a complex sequence environment has made DNA one of the most widely used molecules for programmed molecular self-assembly. DNA has been used to generate discrete nanoscale objects in two- and three-dimensions (1–7), to perform computations (8–12), and to organize biological and non-biological materials (13–17). The DNA nanotechnology field originated from the idea that 3D DNA crystals could be used as molecular scaffolds to determine protein structures (18) and creating periodic 3D DNA arrays has remained one of the major challenges of the field.

One of the difficulties faced in designing DNA crystals comes from the need to overcome limitations of the Watson–Crick duplex. Because the duplex is inherently linear on short length scales, all DNA crystal designs must include some type of branching motif to propagate the lattice into 3D. To date, only one crystal design, based on tensegrity triangles, has been used to form crystals of a continuously base paired DNA lattice from entirely Watson–Crick base pairs (5). Predictable non-canonical base pairing motifs have been envisioned as one way to provide greater structural diversity in DNA crystals, and several such structures have been described (19–21). Additionally, non-canonical DNA motifs including the i-motif, G-quadruplexes and the A-motif continue to find more and diverse uses in DNA nanotechnology (22–26).

Successful crystal designs containing non-canonical base pairs have relied on parallel-stranded (ps) base pairing motifs. Both d(GGA•GGA) and d(CGA•CGA) parallel motifs have been used (19,21). Significantly, both of these motifs have also been observed in solution studies. The d(GGA) motif forms as a pH-independent interlocked arrowhead motif in d(GGAGGAT) (27). The pH-dependent d(CGA) has been observed in multiple sequence contexts and has been characterized as a strong inducer of ps duplexes through a cytosine N3 protonation-dependent mechanism (28). The structurally equivalent d(CGAA) motif (which contains an additional parallel symmetric N1–N6 A–A pair) has been observed in several crystal structures (21,29) and has been verified as a pH-dependent motif in DNA crystals (21). Importantly, all of these ps duplex motifs share a common structural feature: they all exhibit interstrand stacking at the GA dinucleotide steps.

d(GA)_n_ repeat sequences were originally shown to form ps homo duplexes in solution (30). The solution structures of d(CGA) containing duplexes first showed that these repeat sequences contained symmetric sugar-edge N1–N3 G–G pairs followed by Hoogsteen N6–N7 A–A pairs (28). This unique base pairing arrangement results in adjacent nucleobases being stacked with the purine from the partner strand. In solution studies, this interstrand stacking was readily identifiable by the G(H8) to A(H2) Nuclear Overhauser Effect (NOE) crosspeaks (28). This interstrand stacking was subsequently identified as a common feature observed in ps d(GGA) and d(CGA) crystal and solution structures (27,28,31,32). Though solution thermodynamic measurements on these specific ps motifs have not been performed, it was recognized early on that the interstrand stacking was likely a key feature in the duplex stability (28).

Here we describe the crystal structures of a DNA 11-mer that forms eight symmetric ps base pairs. Each oligonucleotide strand forms a ps duplex with a crystallographically equivalent strand, resulting in the first example of a DNA crystal structure that contains four different symmetric homo base pairs. Two of these ps duplexes are coaxially stacked in a head-to-head orientation on equivalent C–C(+) base pairs that is structurally equivalent to the stacking interactions in the DNA i-motif. Remarkably, these two duplexes are effectively locked together as a tetraplex through intercalation of the 5′-most A–A base pairs between adjacent G–G pairs in the opposite duplex. The intercalation region forms a new type of DNA tertiary structural motif with similarities to the i-motif, but that is also structurally isomorphous with GA steps observed in other ps duplexes. Nuclear magnetic resonance (NMR) solution studies and native gel electrophoresis confirmed duplex formation in solution and suggest that the tetraplex may also form in solution. Finally, we show that internal d(GAT) sequence can be replaced by d(GAC), which also yields similar crystals. This indicates that ps d(GAY) containing duplexes may be a useful motif for designing DNA crystals.

## MATERIALS AND METHODS

### Synthesis, purification and crystallization

DNA oligonucleotides were synthesized using standard phosphoramidite chemistry on an Expedite 8909 DNA Synthesizer (Perseptive BioLabs) with reagents from Glen Research (Sterling, VA). One micromole syntheses were purified by 20% denaturing polyacrylamide gel electrophoresis, followed by electroelution, ethanol precipitation and dialysis against deionized water. Native DNA oligonucleotides were crystallized by mixing 0.5 μl of 300 μM DNA solution with 0.5 μl of crystallization solution (100 mM BaCl_2_, 20% MPD and 30 mM sodium cacodylate pH 5.5) in a sitting drop pedestal, with 300 μl of 20% MPD in the well reservoir. Brominated derivatives were crystallized by mixing 0.5 μl of 300 μM DNA solution with 0.5 μl of crystallization solution (100 mM MgCl_2_, 5% PEG400 and 30 mM sodium cacodylate pH 7.4, 8 mM cobalt hexammine) and equilibrated against 300 μl of 20% PEG400 in the well reservoir. Crystals appeared after 1 day and grew as hexagonal plates to a maximum dimension of 100 μm in diameter.

### Data collection and structure determination

Both native and derivative crystals were removed from the drops by nylon loops and plunged directly into liquid nitrogen without additional cryoprotection. Diffraction data were collected at the Advanced Photon Source, Argonne National Laboratory, Beamline 24-ID-E. Data were collected using 0.2 degree rotation angles in shutterless mode, with the exposure time of 0.5 s.

Indexing and integration were performed with MOSFLM (33). For the derivative, initial phases were determined by SAD, using HysS for bromine substructure determination and RESOLVE for density modification in Phenix (34). Electron density maps from the initial SAD phases were sufficient to manually build a model of the derivative structure in Coot (35). Phases from the completed SAD-phased derivative structure were used to calculate initial electron density maps for the native oligonucleotide. The models were built in Coot, followed by refinement in Phenix. Both the native and derivative models were run through the PDB-REDO pipeline (36) following completed refinement in Phenix. The R-factors and geometry were improved for the native structure after the application of single group TLS refinement and 10-fold cross-validation routines of PDB-REDO. Refinement statistics are given in Table 1. Structure factors and coordinates have been deposited in the Protein Data Bank with accession IDs 4RIP and 4RIM.

**Table 1.** Data collection and refinement statistics

	BrU3, BrU8	Native
**Data collection**		
Space group	P6_2_22	P6_2_22
Cell dimensions		
*a, b, c* (Å)	26.4, 26.4,166.5	25.3 25.3 167.8
α, β, γ (°)	90, 90,120	90, 90, 120
Resolution (Å)	55.0–2.03 (2.14–2.03)	50.00–2.07(2.2–2.07)
*R*_merge_	0.063 (0.196)	0.076 (0.35)
*I* / σ*I*	2.93 (3.6)	2.2 (1.8)
Completeness (%)	99.9 (99.9)	99.8 (99.8)
Redundancy	9.5 (9.8)	7.7 (8.2)
Wavelength (Å)	0.9194	0.9720
**Phasing**		
Atom/Sites	Br/2	
FOM	0.38	
FOM, DM	0.57	
**Refinement**		
Resolution (Å)	55.0–2.1 (2.17–2.10)	50–2.3 (2.3–2.36)
No. reflections	3805 (344)	3195 (189)
*R*_work_ / *R*_free_	0.23(0.25)/0.23 (0.28)	0.30 (0.32) /0.30 (0.44)
No. atoms		
DNA	207	199
Water	25	0
*B*-factors		
DNA	31.28	49.51
Water	28.90	N/A
R.m.s deviations		
Bond lengths (Å)	0.007	0.009
Bond angles (°)	1.659	1.524

*Values in parentheses are for highest-resolution shell.

### Nuclear magnetic resonance

NMR data were acquired on two NMR spectrometers, a Bruker Avance III HD 800-MHz spectrometer equipped with a CPQCI cryoprobe, and a Bruker Avance III 600-MHz spectrometer with a CPTCI cryoprobe. The native DNA oligonucleotide NMR samples were prepared at 500 μM in 30 mM sodium cacodylate, pH 4.5, containing 100 mM NaCl, 50 mM MgCl_2_ and 7% D_2_O. The assignments of oligonucleotide base protons, ribose protons and NOE signals were obtained from a combination of 2D-TOCSY (TOtal Correlation SpectroscopY) and 2D-NOESY (Nuclear Overhauser effect spectroscopy) experiments acquired at 285K. Mixing time was set to 120 ms in 2D-TOCSY experiment and 250 ms in 2D-NOESY.

### Native gel electrophoresis

Native gel electrophoresis was performed using native oligonucleotides at 600 μM, incubated overnight with 50 mM Robinson-Britton (RB) buffer (50 mM CH_3_COOH, 50 mM H_3_BO_3_, 50 mM KH_2_PO_4_ pH 6.25) and varying concentration of MgCl_2_ (0, 50, 100, 200 mM). These were mixed with 1 μl of 100% glycerol and loaded on a 13% polyacrylamide gel, pre-equilibrated with running buffer containing 50 mM RB buffer (pH 6.25), 10 mM MgCl_2_ and 100 mM NaCl. For controls, 1 μl of 300 μM native oligonucleotide or 8000 Da oligonucleotide were mixed with 1 μl denaturing buffer (7 M urea, 20 mM ethylenediaminetetraacetic acid, 2 mM Tris pH 7.5, 0.17% (w/v) Xylene cyanol, 0.05% (w/v) Bromophenol blue) prior to loading. Gels were run for 3 h (10V/cm) in an ice bath at 4°C, and stained in 1× SYBR Gold solution (Life Technologies) in water for 20 min.

## RESULTS & DISCUSSION

### Overview and crystal packing

The native and bromine derivative crystals were isomorphous with respect to space group and unit cell dimensions, and have similar overall structures. However, the derivative crystals diffracted to higher resolution and had better overall refinement statistics (Table 1). For these reasons we have chosen to describe the derivative structure except where noted. Complete torsion angle, base pair and base pair step parameters for both structures are given in Supplementary Tables S1 and S2.

**Figure 1.**
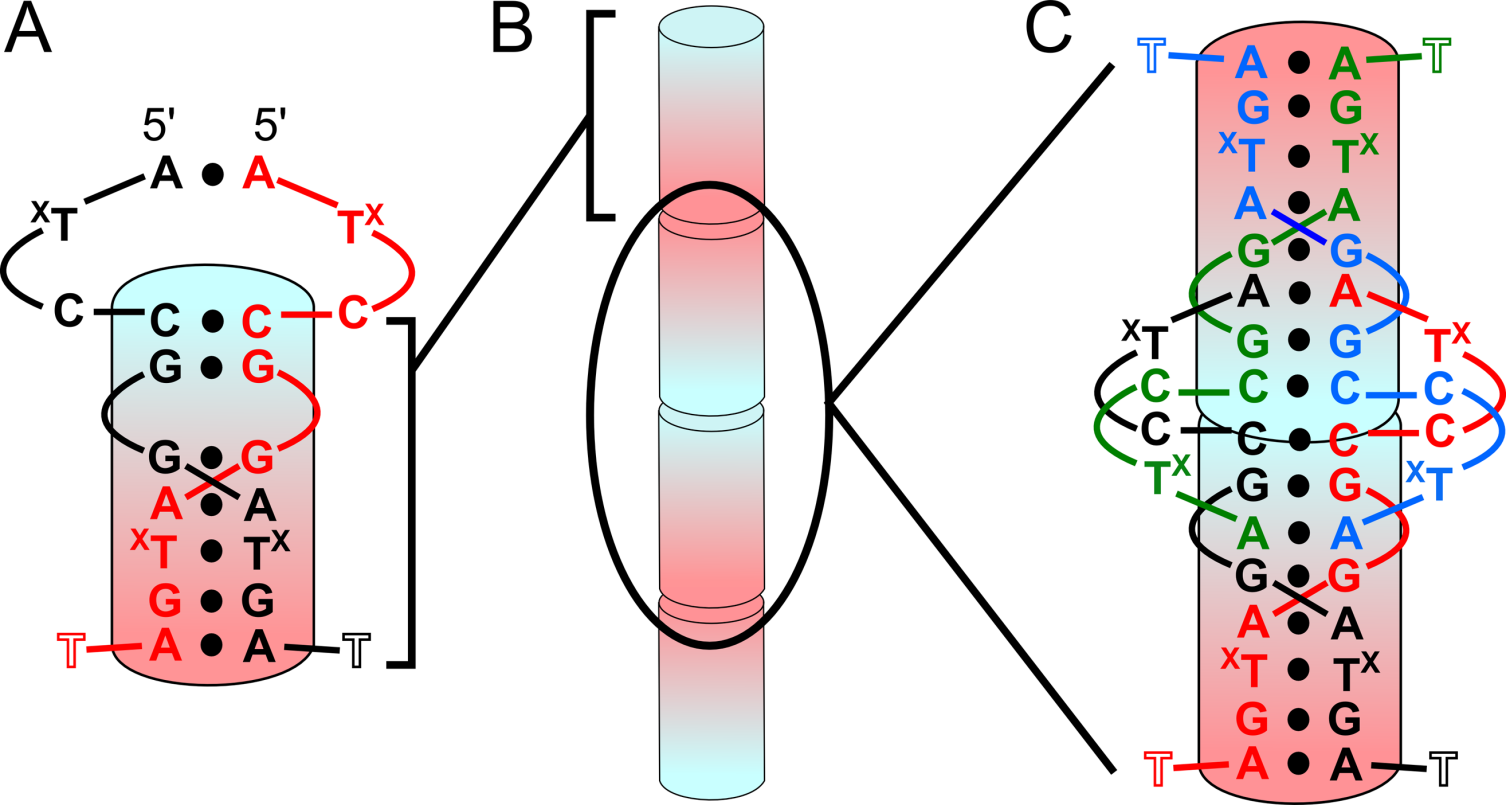
Secondary structure and crystal packing. (**A**) Secondary structure of interactions formed between two identical DNA strands. The ps duplex region is shown as the shaded cylinder going from 5′ (blue) to 3′ (red). T^X^ represents dT or 5-Br dU residues. T11 was mostly disordered. (**B**) Representation of coaxial 5′–5′ and 3′–3′ stacking of duplexes along the crystallographic *c* cell axis. (**C**) Secondary structure of two duplexes at the 5′–5′ interface with each of the four strands colored differently. The two duplexes stack on the C4–C4 pairs.

The DNA oligonucleotide crystallized with one molecule in the asymmetric unit. Thus, all DNA strands in the crystal are identical in conformation. Each strand interacts with a partner strand through ps homobase pairing (Figure 1A). These ps duplexes are coaxially arrayed along the crystallographic *c* cell axis through 5′-to-5′ and 3′-to-3′ stacking interactions between C4–C4 pairs and A10–A10 pairs, respectively (Figure 1B and C). C2 and ^Br^U3 are unpaired and their nucleobases are perpendicular to the duplex helical axis where they stack with C2 and ^Br^U3 nucleobases from neighboring duplexes (Supplementary Figure S1). These type of stacking interactions are reminiscent of pyrimidine nucleobase stacking interactions observed in a number of nucleic acid crystal structures (37–39) and may represent a general nucleic acid crystal packing motif. The major differences between the derivative and native structures are in these pyrimidines positions (see below).

### Homoduplex region

**Figure 2.**
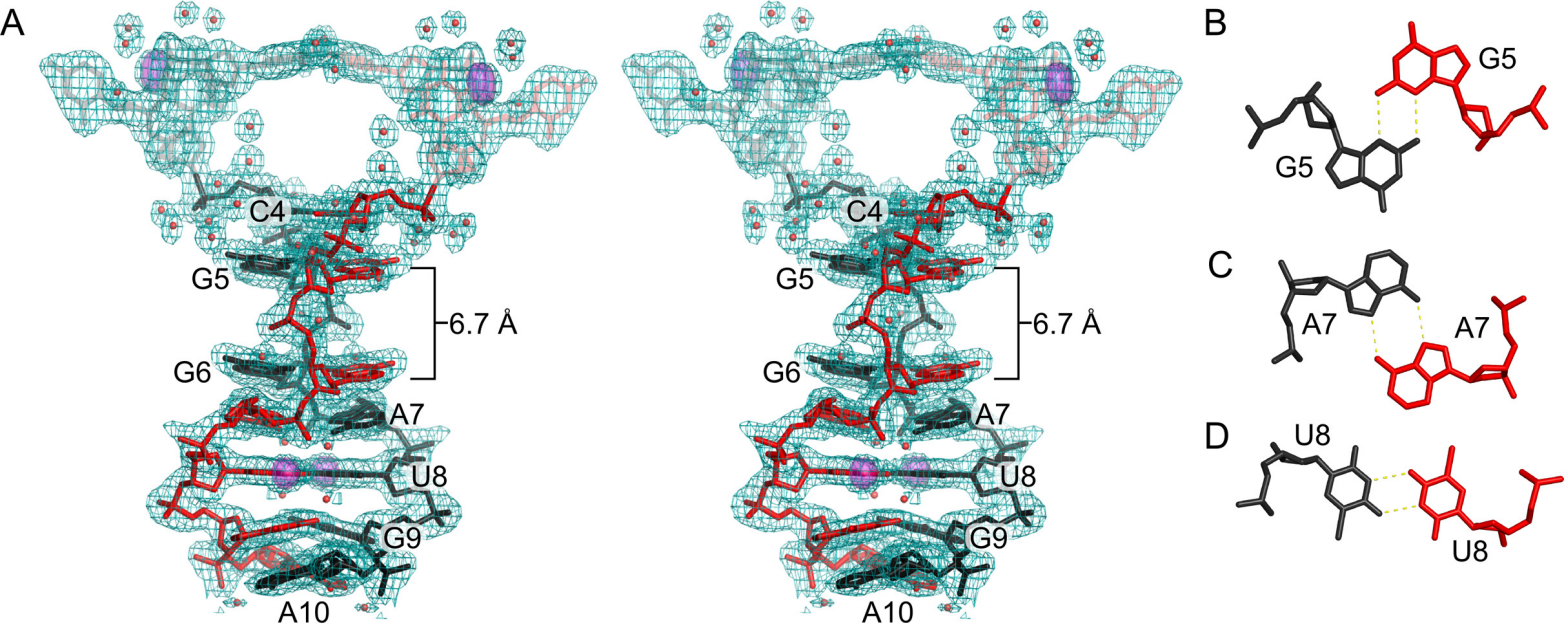
Parallel-stranded duplex. (**A**) Stereoview of residues 1–10 from two monomers that form a ps duplex shown with 2mFo–DFc electron density contoured at 0.75 σ. Parallel-stranded duplex residues are labeled. Residues outside the duplex region (A1–U3) are semi-transparent. Anomalous difference electron density contoured at 5 σ (violet surface) corresponds to bromine atoms used for phasing. Water molecules are shown as red spheres. The gap between residues G5 and G6 is 6.7 Å. (**B–D**) Individual base pairs showing hydrogen bonding between identical residues. (B) N2–N3 sugar edge interactions between G5–G5, G6–G6 and G9–G9 homo base pairs. (C) N6–N7 Hoogsteen interactions between A7–A7 and A10–A10. (D) Symmetric N3–O4 hydrogen bonding observed for U8–U8 homo base pair.

Parallel-stranded base pairing between C4 and A10 of two strands forms a continuous ps homoduplex (Figure 1A). Similar to other homoduplex structures containing d(GGA) and d(CGA) ps interactions (27–29,31), this parallel duplex region forms a right-handed helix (Figure 2A). C4–C4 base pairing occurs through well characterized symmetric interactions of the Watson–Crick faces, resulting in three hydrogen bonds that require hemiprotonation at the N3 position. Interestingly, derivative crystals were grown at neutral pH, suggesting that N3 protonation can occur at non-acidic pHs. This is in contrast to previous DNA structures we have determined (21) in which the parallel CG step could transition between two parallel base pairs or a single C–G–G–C quadruplex in a pH-dependent manner. However, this is consistent with several other observations (40) and may be dependent on a number of factors, including the local concentration (41). G5, G6 and G9 are in the *anti* conformation and form homo base pairs through symmetric N2–N3 sugar-edge interactions (Figure 2B). For G5 and G6, N2 is in hydrogen bonding distance with both N3 and O4′ of their partners. Notably, there is a 6.7 Å gap between G5 and G6 nucleobases in the homoduplex (Figure 2A). A7 and A10 are also in the *anti* conformation and form homo base pairs through symmetric N6–N7 Hoogsteen interactions (Figure 2C). A7 makes an additional cross-strand hydrogen bond contact with the ^Br^U8 nucleobase (N6–O4) of the partner strand, while water molecules form bridging hydrogen bonds between N6 and a non-bridging phosophate oxygen of the base pair partner for both A7 and A10 pairs. The ^Br^U8 pairing is through symmetric N3–O4 hydrogen bonding (Figure 2D). ^Br^U8 also has shorter (2.86 Å) hydrogen bonding distance than the homopurine interactions (3.03 and 3.45 Å for A7 and G9, respectively). The last nucleotide, T11, is mostly disordered with only the phosphate present in the electron density.

One of the notable features of the ps duplex region is the difference in base pair buckle and propeller parameters between the purine and pyrimidine homo pairs. As indicated in Supplementary Table S2, the two pyrimidine homo base pairs have small propeller and buckle angles with respect to the duplex axis, while all the purine pairs have larger buckle and propeller angles. Additionally, the structure suggests some degree of variability of the purine buckle and propeller. The internal A7 base pair has a high buckle angle (−30.4°) and moderate propeller (−17.9°). The identical A10 base pair has lower buckle (−10.5°) and high propeller (−40.7°). This is likely due to the differences in the flanking pairs, as A7 is followed by the more planar ^Br^U8, while A10 is flanked by purines on both sides (G9 and A10 from another duplex; see Figure 1).

Two GA steps stabilize the duplex through interstrand stacking interactions. The stacking interactions between G6 and A7 and G9 and A10 are typical interstrand stacking for the ps GA steps, typified by the large helical twist parameter (Supplementary Table S2). Superposition comparison of the G6–A7 and G9–A10 steps to each other indicate that these steps are highly similar, with all-atom RMSD of 0.719 Å and they are virtually identical to GA steps from other crystal structures (29). The structural similarity between these dinucleotide steps supports the importance of the interstrand stacking interactions in stabilizing this type of non-canonical structure, while also suggesting that this motif may be capable of forming in other sequence contexts.

Interestingly, this GA interstrand stacking motif has not yet been observed in RNA structures. Examination of DNA crystal structures containing parallel GA steps suggests that in RNA the ps motif would likely be destabilized by steric clashes between the 2′ OH of the guanosine and the stacked adenosine partner. However, this is based on all current observations of the sugar pucker of this G being C2′-endo.

### 5′-to-5′ stacking and intercalation forms a tetraplex

Base stacking between strands at duplex ends is commonly observed in DNA crystal structures. In many cases these crystal contacts are non-specific with respect to the base identities, though depending on crystal packing there may be geometric restrictions. These non-specific stacking interactions often organize shorter helical segments into pseudo-infinite helices. This is also true for the 3′-to-3′ stacking observed in this structure, in which four crystallographically equivalent A10 residues interact exclusively through nucleobase stacking (Supplementary Figure S2). In contrast, the 5′-to-5′ stacking results in more extensive interactions.

**Figure 3.**
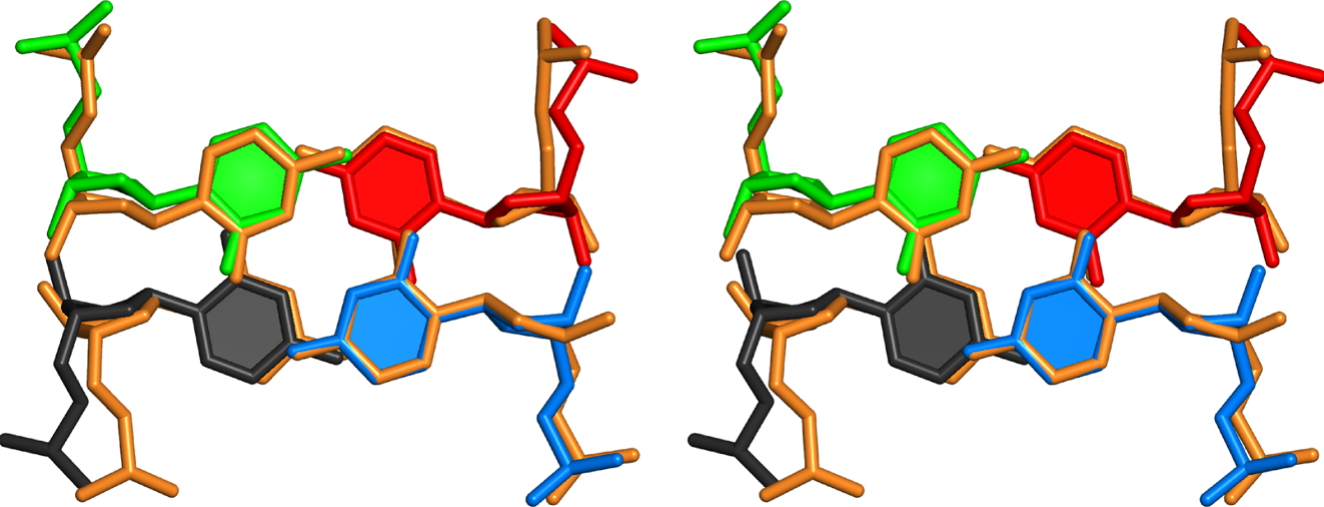
5′–5′ stacking. Stereo view of orthogonally stacked C4–C4 homo-base pairs (black, red, blue, green) of two ps homoduplexes. These 5′–5′ stacking interactions are superpositioned with a single step of an i-motif to illustrate the structural similarity (PDB ID: 1CN0; orange).

**Figure 4.**
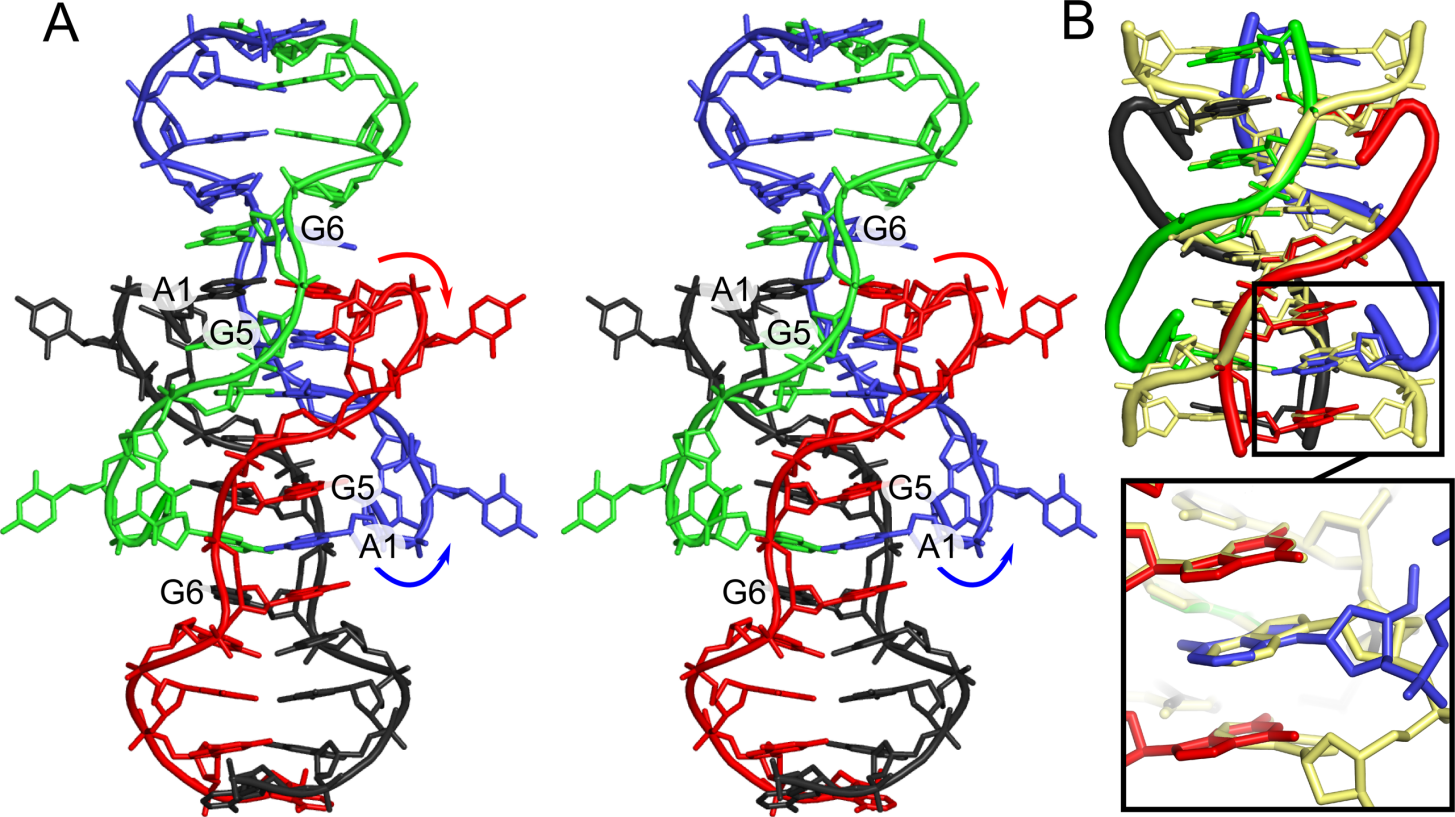
An intercalation-locked tetraplex. (**A**) Stereo view cartoon and stick representation of a tetraplex with all four strands colored differently. Arrows indicated flipping of A1 residues with respect to ps duplex region. A1–A1 base pairs are intercalated between G5–G5 and G6–G6 pairs of opposite duplex, locking the two duplexes into a tetraplex. (**B**) Superposition the 5′–5′ interface and A1 residues with 5′–5′ stacked ps CGAA motifs (yellow) (29). C2 and U3 have been removed for clarity. The motifs are structurally similar, and the intercalated A1 residues superpose with first A–A pair of the GA dinucleotide step from the CGAA motif (inset).

The C4–C4 base pairs of each homo-duplex are orthogonally stacked and are structurally equivalent to one intercalation step of a DNA i-motif (26,42–44) (Figure 3). The intermolecular stacking of two C–C(+) pairs followed by G–G base pairs has been observed in several other crystals structures (21,29) and in solution for the dimeric molecule of d(TCGTTTCGT) (45). However, the 5′–5′ interactions not rely solely on these stacking interactions. Nucleobase intercalation locks the two duplexes together, though surprisingly, it is not cytidine, but adenosine nucleobases that are intercalated. Like A7 and A10, the A1 nucleobases are in the *anti* conformation and base pair through symmetric N6–N7 pairing. However, this residue differs in that its sugar and nucleobase are flipped with respect to the homoduplex axis in a manner analogous to the alternating orientation of residues in the Z-DNA duplex (46). This flipping changes the presentation of functional groups with respect to the parallel-duplex groove, but because both paired A1 residues are flipped, they maintain the N6–N7 symmetric hydrogen bonds. The A1 residues are separated from their own homoduplex region through the unpaired pyrimidines (Figure 1A). This positions them to intercalate between G5–G5 and G6–G6 of the opposite homoduplex (Figure 4A). Thus, the A1 pairs convert the two stacked duplexes into a tetraplex by maintaining continuous base stacking through intercalation.

An important consequence of the A1 directional flipping is that this residue is oriented in the same direction as those of the homoduplex region of the tetraplex partner. Thus, the intercalated A1 is parallel with respect to the G5 and G6 residues that flank it (Figure 4A). This allows for almost identical purine stacking interactions between the intercalated A1 base and the G5 base and the GA steps observed in this (G6–A7, G9–A10) and other GA-containing parallel duplexes (28,29). Superpositioning of the tetraplex intercalation region and the four strands that make up the non-canonical d(CGAA) ps region (29) shows the two stacking motifs are nearly identical (RMSD 0.89 Å for 38 atoms from A1, C4 and G5 residues), with the flipped A1–A1 base pairs being nearly identical to the first A–A pair in the ps d(CGAA) motif (RSMD: 0.197 Å) (Figure 4B). This is a remarkable structural similarity, despite the intercalation region representing a tertiary interaction within a very different sequence environment. This further suggests that G–A interstrand stacking interactions are favorable in a variety of sequence and structural environments.

### Halogen–halogen and halogen bonding interactions stabilize the derivative crystal lattice

We initially crystallized the non-derivatized DNA oligonucleotide, and utilized the ^Br^U3 and ^Br^U8 substitutions for phasing purposes. Though we were capable of collecting data on isomorphous native crystals, we had difficulty refining the structure despite the initial phase estimates indicating a structure very similar to the SAD-phased derivative (Supplementary Figure S3A). The main difference between the native and derivative structures is the locations of extra-helical pyrimidine residues. Specifically, T3 in the native structure is not involved in the same type of crystal stacking contacts as ^Br^U3, but is instead orientated toward the helical axis, allowing it to stack with T3 from the partner strand of the tetraplex, while also forming a hydrogen bond with C4 (Supplementary Figure S3B). Additionally, C2 was nearly completely disordered in the native crystals. This suggests that ^Br^U3 locally stabilizes the pyrimidine packing interactions that orient adjacent tetraplexes in the crystal lattice. Indeed, ^Br^U3 is involved in halogen–halogen interaction with the bromine of ^Br^U3 from and adjacent tetramer (Supplementary Figure S4A). Additionally, the ^Br^U8 5-bromo position makes a potential halogen bond (47,48) with a phosphate oxygen from A7 (Supplementary Figure S4B). The energetic contributions from the attractive halogen–halogen interactions and halogen bonding can be quite significant (48,49). The lower resolution and poorer overall refinement statistics for the native structure is most likely explained by a loss of higher-order lattice structure due to the absence of these halogen interactions.

### Formation of a parallel-stranded duplex in solution

**Figure 5.**
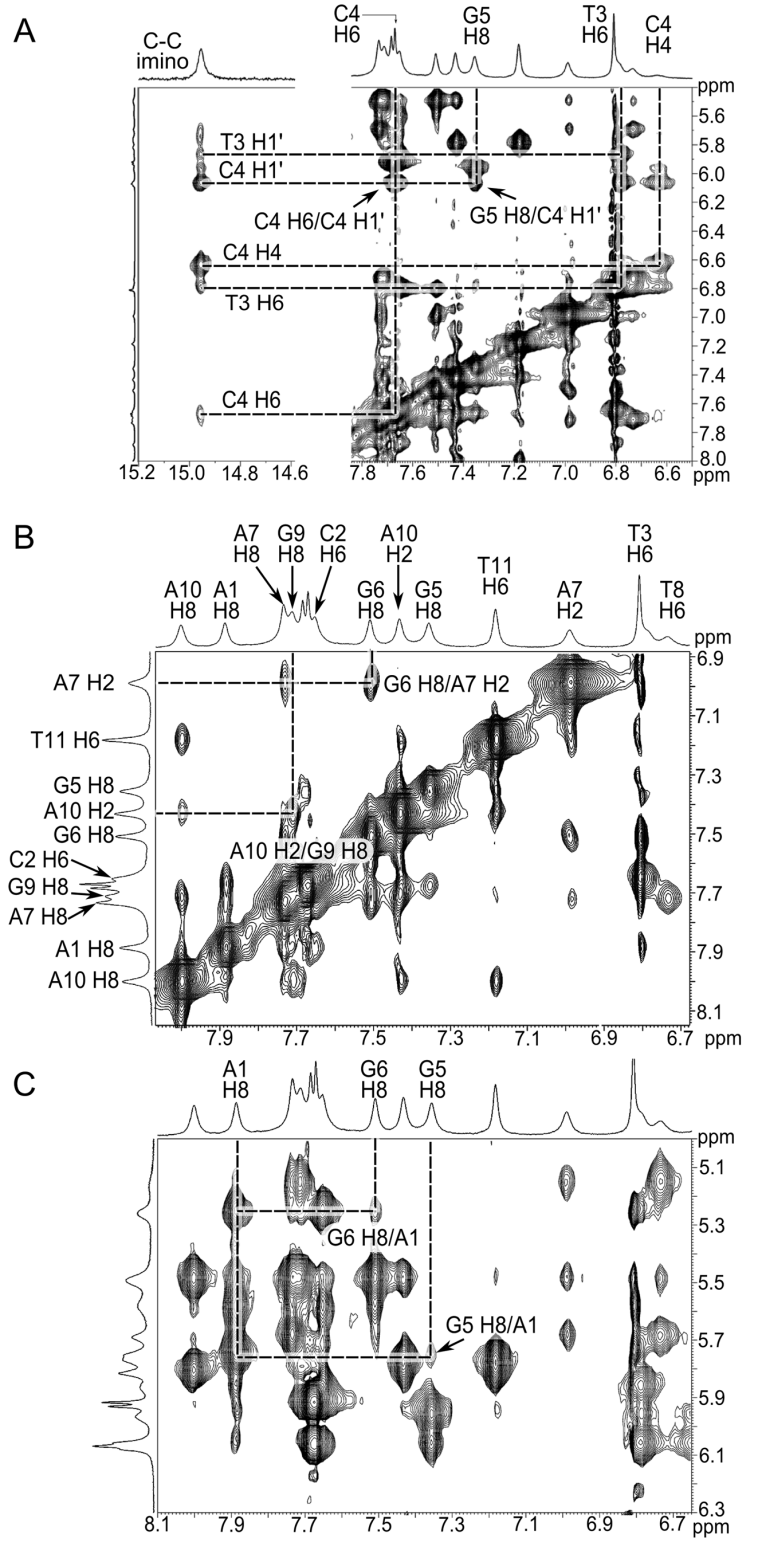
2D-NOESY NMR spectra. (**A**) Characteristic C4–C4(+) imino signal at 14.95 ppm and its identified cross peaks. (**B**) Peak assignments indicating G6–A7 and G9–A10 interstrand stacking in 2D-NOESY NMR spectra, consistent with the ps homo duplex crystal structure. G6 H8 and A7 H2 cross peaks and G9 H8 and A10 H2 are labeled and indicated by intersecting lines. (**C**) 2D-NOESY NMR spectra indicating interactions between A1 and G5/G6. Cross peaks indicate NOE between these residues, however, peak assignments were not complete for the A1 stack so specific protons have not been assigned for A1.

We examined the native oligonucleotide by ^1^H NMR and native gel electrophoresis to determine if the interactions observed in the crystal structure were also present in solution. Several hallmarks in the NMR data strongly suggested the formation of a ps duplex. First, the hemi-protonated C–C(+) observed in the i-motif and in d(CGA) containing structures have a distinct N3 imino proton peak at ∼15 ppm (31). We observed a clear single resonance at 14.95 ppm typical of a C–C(+) pair (Figure 5A) at all temperatures tested (Supplementary Figure S5). Further, we identified both G(H8)–A(H2) NOE crosspeaks for the G6–A7 and G9–A10 dinucleotide steps (Figure 5B). These cross-peaks are consistent with the interstrand stacking observed in the crystal structure and all previous solution structures containing ps GA steps. Interestingly, though we could readily detect NOE cross peaks between the G6 sugar (H2′) and G5 nucleobase (H8), we were unable to detect cross peaks between G5 and G6 nucleobases. This suggests that the nucleobases are not stacked. Consistent with these findings and the crystal structure, we were able to observe several A1 to G5 and A1 to G6 cross peaks (Figure 5C). These peaks were relatively weak, but they suggest interactions similar to the A1–A1 intercalation of the tetraplex.

As an additional examination of the oligomeric state, we performed native gel analysis. Gels run in the absence of Mg^2+^ showed little or no higher molecular weight product relative to a denatured reference sample. However, the addition of Mg^2+^ to the sample prior to loading showed the appearance of a higher molecular weight product that ran at the same molecular weight as a control oligonucleotide with a molecular weight of 8000 (Supplementary Figure S6), suggesting the formation of a dimer. The appearance of the duplex was not greatly enhance when samples were incubated with Mg^2+^ higher than 50 mM. Together, the NMR and native gel analysis results strongly indicate that the oligonucleotide is capable of forming a ps duplex in solution and the NMR data suggests that the duplexes may assemble into tetraplexes.

### Sequence modifications and implications for crystal design

To test if modifications could be made to the crystal lattice, we examined a number of substitutions and expansions of the oligonucleotide sequence. Sequences and crystallization results are summarized in Supplementary Table S3. We anticipated that T to C pyrimidine substitutions in the ps duplex should have little impact on crystal assembly based on structural similarity. Oligonucleotides with the T8C substitution readily crystallized under the same conditions as the derivative and native oligonucleotides, while those that contained both T8C and T11C crystallized, but were of poor quality. None of the oligonucleotides containing a 3′ C diffracted to high resolution (Supplementary Table S2), suggesting that a terminal C–C base pair likely prevents proper 3′–3′ stacking of terminal A residues that may be necessary to maintain lattice packing. Next, we examined how the addition of d(GAY) sequences affected crystallization. The addition of one d(GAT) to the 3′ end of the native oligonucleotide did not provide crystals, however, when the two internal T's were substituted with C's, we obtained diffraction-quality crystals. Replacing all three T's with C's yielded crystals, though these diffracted poorly. Our preliminary analysis of diffraction data from these crystals indicate that both of the T8C substitution and the d(GAC) extension contain a nearly identical tetraplex to the structures described here, despite crystallizing in different space groups and having different numbers of molecules in the asymmetric units (C2 for T8C with four molecules in the asymmetric unit; P6_5_22 for the GAC extension with two molecules in the asymmetric unit). Complete structural description of these oligonucleotides will be presented elsewhere.

The structures described here may provide a new toolkit for designing DNA crystals containing non-canonical motifs. Our results indicate the intercalation-locked tetraplex is a robust structural motif that provides predictability at the tertiary contact level. Further, we have preliminary evidence that d(GAT) and d(GAC) motifs are structurally isomorphous. Thus, these distinct but related motifs could provide sequence specificity for ps duplex assembly in a multiple oligonucleotide assembly process. Robinson and Wang (31) pointed out that ps duplexes of d(GA)_n_ runs could easily slip between strands, and that the addition of the pyrimidines into the ps motif provides greater discrimination during duplex association. Demonstrating that both d(GAT) and d(GAC) sequences can be incorporated in the ps duplex allows for greater complexity in sequence design. These are both important consideration for the programmed assembly of DNA crystals or other 3D DNA nanoassemblies with non-canonical base pairs.

## ACCESSION NUMBERS

PDB IDs: 4RIP and 4RIM.

## SUPPLEMENTARY DATA

Supplementary Data are available at NAR Online.

SUPPLEMENTARY DATA
