# Supplementary material for: An intercalation-locked parallel-stranded DNA tetraplex
Source: Nucleic Acids Res. 2015 Jan 27;43(3):1937–44. doi: 10.1093/nar/gkv033 (PMC4330391; doi:10.1093/nar/gkv033)
Supplement: SUPPLEMENTARY DATA [file supp_43_3_1937__index.html]

An intercalation-locked parallel-stranded DNA tetraplex — SUPPLEMENTARY DATA 

# An intercalation-locked parallel-stranded DNA tetraplex

## SUPPLEMENTARY DATA

**Files in this Data Supplement:**

- SUPPLEMENTARY DATA
