# Supplementary material for: An intercalation-locked parallel-stranded DNA tetraplex
Source: Nucleic Acids Res. 2015 Jan 27;43(3):1937–44. doi: 10.1093/nar/gkv033 (PMC4330391; doi:10.1093/nar/gkv033)
Supplement: SUPPLEMENTARY DATA [file supp_gkv033_nar-03517-f-2014-File007.pdf]

Supplementary Material For:

An intercalation-locked parallel-stranded DNA tetraplex

Shailesh Tripathi, Daoning Zhang & Paul J. Paukstelis

University of Maryland

Department of Chemistry & Biochemistry

Center for Biomolecular Structure & Organization

Maryland Nanocenter

College Park, MD 20742

paukstel@umd.edu

Table S1 DNA Torsion Angles*

	base	χ	anti/syn	α	β	γ	δ	ε	ζ	pucker
**Bromo**	A1	-131.9	anti			-161.3	150.2	-109.1	82	C2'-endo
	C2	-96.3	anti	65.3	165.7	44.3	130.8	-100.7	-95.1	C1'-exo
	U3	-159.8	anti	-82.5	-142.9	59.7	149.4	-125.8	-80	C2'-endo
	C4	-120.6	anti	-54.7	-147.2	54.3	143.7	-161.9	-85	C2'-endo
	G5	-80.8	anti	-61.7	173.4	41.3	143.7	-160.8	-68	C2'-endo
	G6	-76.1	anti	153.8	111.9	173.8	137.3	-139.1	148	C2'-endo
	A7	-171.7	anti	-77.3	134.6	58.9	83.9	-172.9	-22.2	C4'-exo
	U8	-128.4	anti	-115.3	172.7	90.6	129.2	-168.5	-75	C1'-exo
	G9	-98.6	anti	-62.1	172.2	51.7	130.2	-120.2	137.3	C2'-endo
	A10	-170.4	anti	98.5	-145.2	-164.1	151.6	-117.1		C3'-exo
**Native**	A1	-125.2	anti			-103.4	153.3	-145.4	134	C2'-endo
	C2	N/A	N/A	-113.3	117.4	166.1	151.2	-75	105.5	C3'-exo
	T3	-98.2	anti	14.3	149.6	-107.1	158.5	-154.1	-171.4	C3'-exo
	C4	-119.5	anti	-58.7	169.7	44.4	139	-172.1	-66.9	C2'-endo
	G5	-79.9	anti	-78.2	179.3	50.5	135.8	-165.3	-70.1	C2'-endo
	G6	-81.1	anti	162.8	109	175.7	133.1	-144.7	149.4	C2'-endo
	A7	-164.3	anti	-71	133.3	54.5	88.6	-146.3	-62.4	C3'-endo
	T8	-118.8	anti	-60.2	167.6	63.7	141.7	179.3	-75.7	C2'-endo
	G9	-87.9	anti	-74.6	-170.4	57	138.5	-128.2	160.7	C2'-endo
	A10	-160.6	anti	-71.7	148	49.3	89.9	49.8		C4'-exo

* Torsion angles calculated with X3DNA (1).

Table S2. Base pair and base pair step parameters.

Base pairs[a]	bp	Shear	Stretch	Buckle[c]	Propeller[c]
	A+A	5.59 (5.75)	5.11 (4.96)	-9.42 (-14.89)	-23.33 (-23.80)
	C+C	1.85 (2.1)	1.38 (1.17)	-0.13 (-0.26)	-5.42 (-2.57)
	G+G	3.38 (3.22)	8.05 (8.07)	10.48 (10.44)	28.17 (25.38)
	G+G	3.2 (3.36)	8.05 (7.98)	17.2 (16.26)	21.81 (20.90)
	A+A	6.37(5.89)	5.59 (5.06)	-30.43 (-26.29)	-17.85 (-14.45)
	U+U	1.64 (1.66)	1.6 (1.44)	0.09 (7.57)	1.91 (2.64)
	G+G	3.58(3.54)	8.74 (8.08)	8.37 (6.32)	27.14 (31.57)
	A+A	5.16(5.41)	5.41 (5.15)	-10.46 (-11.31)	-40.69 (-36.62)

Base pair steps[b]	step	Rise	Twist
	AC/CA	9.63 (9.92)	28.84 (24.27)
	CG/GC	4.2 (4.12)	14.8 (11.62)
	GG/GG	6.78 (6.75)	-4.65 (-8.71)
	GA/AG	2.64 (2.89)	91.44 (94.21)
	AU/UA	3.85 (3.72)	10.69 (12.1)
	UG/GU	4.47 (4.49)	9.8 (10.0)
	GA/AG	1.68 (1.86)	95.57 (97.32)

a. Values for the brominated derivative calculated with X3DNA (1), with only non-zero parameters shown. Values in parentheses are for the equivalent pair in the native structure.

b. Values for the brominated derivative calculated with X3DNA using CEHS parameters (2), with values in parentheses for the equivalent step of the native structure. Base pair step parameters are identical to helical parameters in the case of a perfectly symmetrical parallel duplex. Only non-zero parameters are shown.

c. Positive and negative angles were assigned based on the planar base vector pointing toward the 5' (+) or 3' (-) end of the parallel duplex.

Table S3. Crystallization and diffraction quality of alternate oligonucleotides

Oligonucleotide	Crystals*	Diffraction limit (Å)
ACTCGGATGAT (native)	Yes	2.0
ACTCGGACGAT	Yes	2.1
ACTCGGACGAC	Yes	> 6
ACTCGGACGAA	Yes	Not tested
ACTCGGATGATGAT	No	N/A
ACTCGGACGACGAT	Yes	2.6
ACTCGGACGACGAC	Yes	> 6

* Only conditions in Materials & Methods were tested

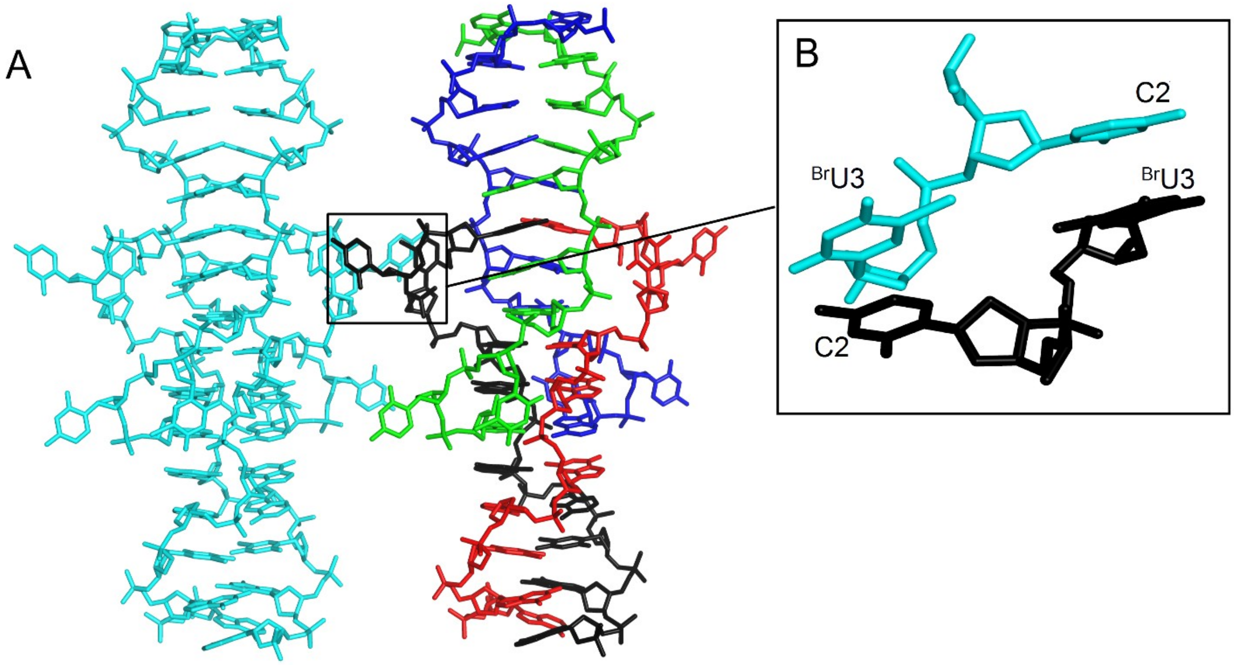

Figure S1. Crystal lattice packing. **A**. Interaction between adjacent units through C2-^{Br}U3 stacking interaction. Two adjacent units are shown. **B**. Close up view of C2- ^{Br}U3 stacking interaction.

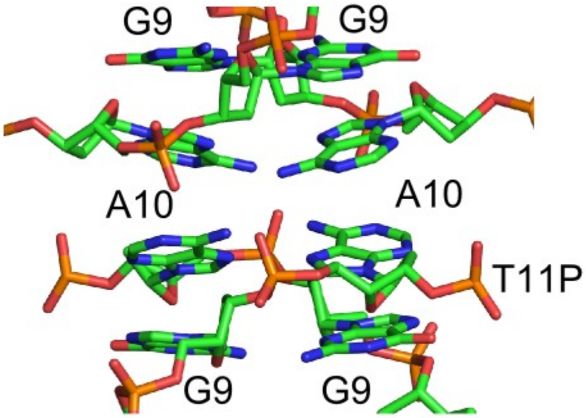

Figure S2. 3'-to-3' stacking stabilizes the co-axially stacked duplex units along crystallographic c-axis. All four adenines (A10) are related by crystallographic symmetry. T11 was mostly disordered, with only the phosphate present in the electron density.

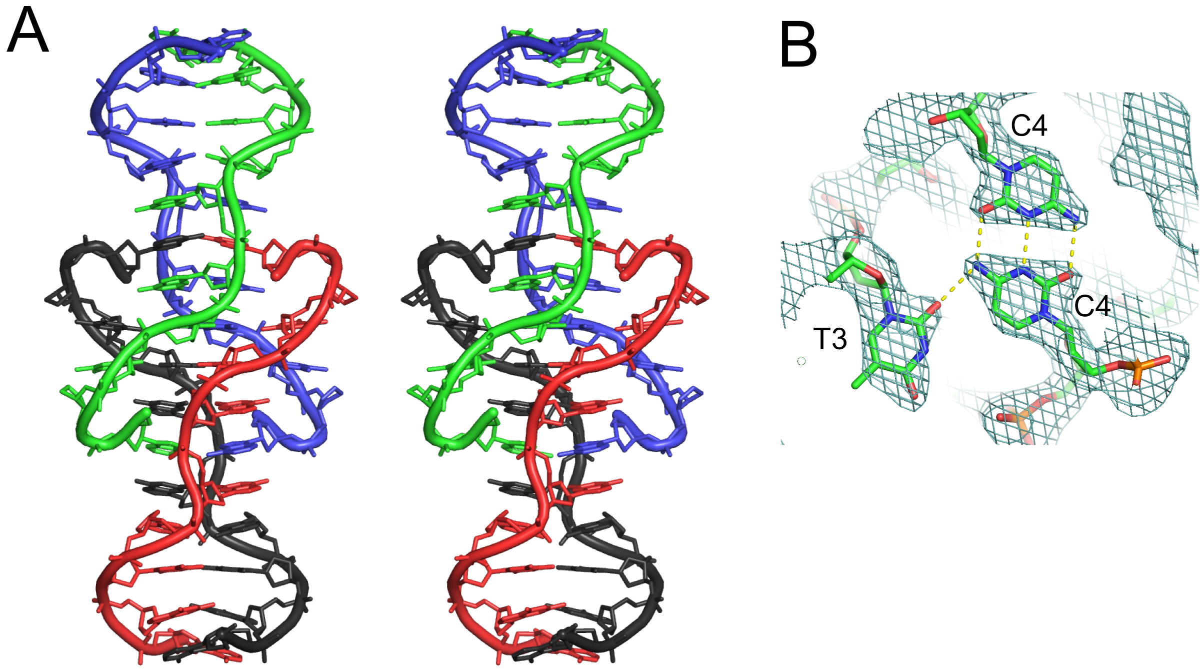

Figure S3. Native tetraplex structure. **A**. Stereo view of intercalated ps tetraplex structure of native oligonucleotide with each strand colored differently. The C2 nucleobase was disordered in this structure. **B**. T3-C4 hydrogen bond.. SigmaA-weighted $2F_o$-F_c electron density is calculated at 1.5 σ. O2 of T3 is involved in hydrogen bonding interaction (2.86 Å) with N4 of C4 of its partner strand.

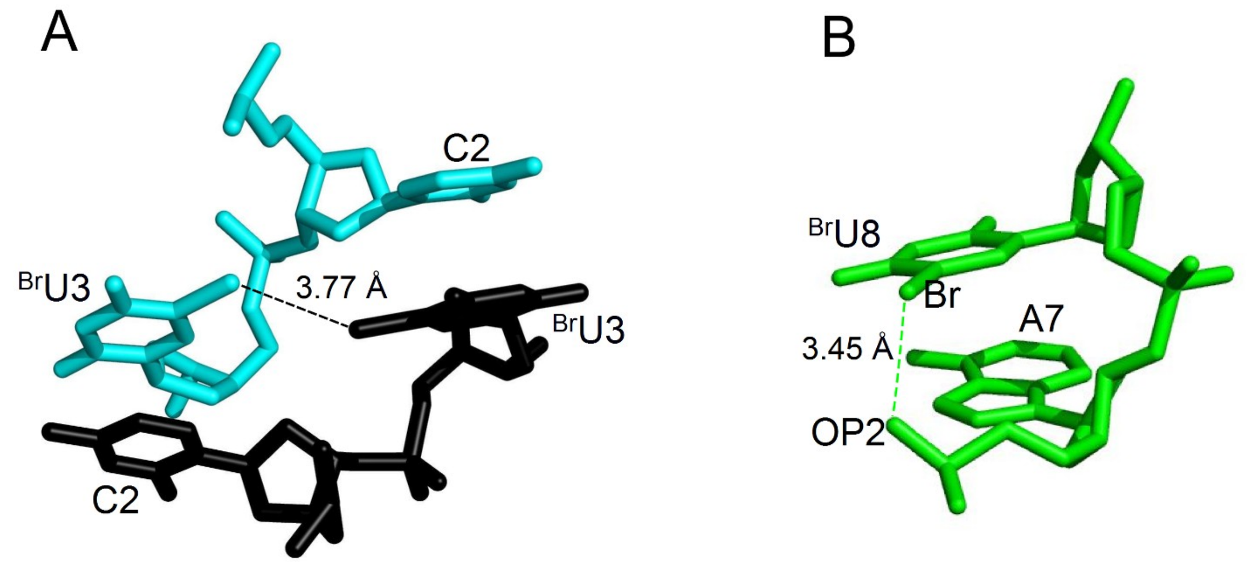

Figure S4. Halogen-halogen and halogen bonding interactions. **A**. Bromines of ^{Br}U3 from adjacent tetraplex units are oriented towards each other and are involved in halogen-halogen attractive interactions with a distance of 3.77 Å. **B**. Bromine of ^{Br}U8 is involved in halogen bonding interaction with non-bridging oxygen (OP2) of A7 (3.45 Å).

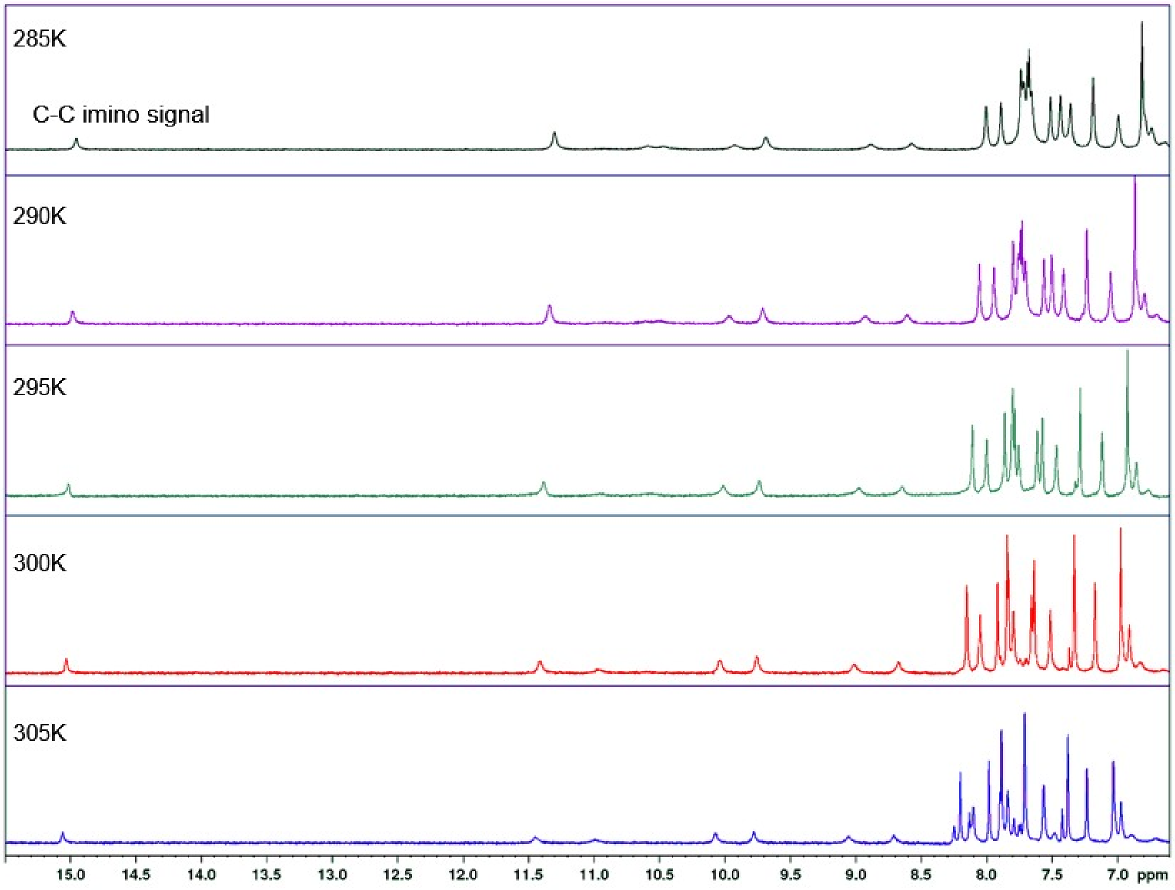

Figure S5. One dimensional proton NMR spectra of native oligonucleotide. Distinct C-C(+) peak appeared near 15 ppm at all temperatures. The number of peaks in the region 8.5 to 6.5 ppm increased at higher temperature, possibly indicating presence of several temperature-dependent conformations.

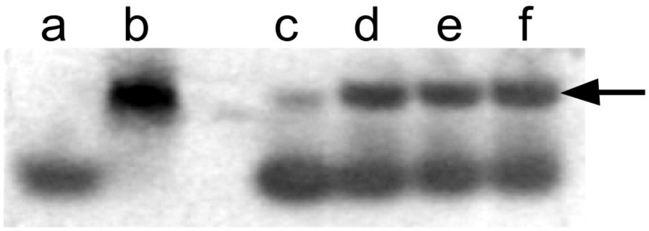

Figure S6. Native gel electrophoresis. Lanes a and b, native DNA 11-mer and a 8000 Da oligonucleotide, respectively, incubated with denaturing buffer. Lane c to f, native DNA 11-mers incubated in Robinson-Britton buffer with 0, 50, 100 and 150 mM $MgCl_2$, respectively. The arrow head indicates the appearance of the major second band of approximately 8000 Da.

Supplementary References

1. Lu,X.-J. and Olson,W.K. (2008) 3DNA: a versatile, integrated software system for the analysis, rebuilding and visualization of three-dimensional nucleic-acid structures. *Nat. Protoc.*, **3**, 1213–1227.

2. El Hassan,M.A. and Calladine,C.R. (1995) The assessment of the geometry of dinucleotide steps in double-helical DNA; a new local calculation scheme. *J. Mol. Biol.*, **251**, 648–664.
